# The latent profiles of parent and peer attachment through the lens of adolescents: differences in life satisfaction, resilient mindset, basic psychological needs, and relative deprivation

**DOI:** 10.3389/fpsyg.2026.1770861

**Published:** 2026-03-16

**Authors:** Raziye Yüksel Doğan, Haktan Demircioğlu

**Affiliations:** Department of Child Development, Faculty of Health Sciences, Hacettepe University, Ankara, Türkiye

**Keywords:** adolescence, adolescent outcome indicators, latent profile analysis, parent attachment, peer attachment

## Abstract

**Purpose:**

This study employs a person-centered approach to explore the latent profiles of adolescents’ parent and peer attachment and to reveal potential disparities between their levels of life satisfaction, resilient mindset, perceived basic psychological needs satisfaction and frustration, and relative deprivation by their attachment profiles.

**Methods:**

The cross-sectional exploratory study included 2,322 adolescents aged 14–18 attending secondary schools in Ankara, Türkiye (*M*_age_ = 15.91, *SD* = 1.13; females = 57.1%). We collected the data using the Inventory of Parent and Peer Attachment, the Satisfaction with Life Scale, the Resilient Mindset Scale, the Basic Psychological Need Satisfaction and Frustration Scale, and the Relative Deprivation Scale. Latent profile analysis was employed to identify attachment profiles. Additionally, Welch’s ANOVA was conducted to examine differences in adolescent outcome indicators across the identified attachment profiles. Pairwise comparisons were then made using the Games-Howell Test due to the non-homogeneity of the variances. Effect sizes were reported as eta squared (η^2^).

**Results:**

Our analysis yielded seven latent profiles of parent and peer attachment during adolescence, and we compared the outcome indicators specified in the study by these profiles. Accordingly, we established that life satisfaction, resilient mindset, basic psychological needs satisfaction and frustration, and perceived relative deprivation showed significant differences by participating adolescents’ attachment profiles. Our findings demonstrated that the identified latent attachment profiles exhibited not only statistical but also theoretical and practical validity and relevance.

**Conclusion/practice implications:**

This study sought to elucidate the complex nature of attachment hierarchies during adolescence and highlight the protective role of secure attachment to multiple figures. Our findings highlight the potential importance of developing targeted, customized strategies for adolescents with different attachment profiles while promoting optimal development through attachment-based approaches.

## Introduction

Adolescence is a distinct period characterized by physical, sexual, cognitive, social, and emotional growth, representing one’s transition from childhood to adulthood (Dahl et al., 2018; Kulaksızoğlu, 2017). Several indicators in this phase can affect the adolescent’s optimal development, psychosocial adaptation, and overall well-being while their familial and peer dynamics experience substantial normative transformations (Santrock, 2017; Steinberg, 2007). One such indicator is parent and peer attachment. Allen and Land (1999) aired their remarkable observation regarding adolescent attachment 25 years ago:

*“In several aspects, our understanding of attachment during adolescence is substantial; but, in other respects, it remains quite limited... Attempts to assess attachment in adolescence must inevitably confront the question of what attachment is at this stage of life and what function it serves.”*

Despite the cumulative increase in our understanding of adolescent attachment, substantial gaps remain in the literature concerning the examination of multiple indicators of parent and peer attachment during adolescence and their potential impacts on adolescents’ optimal development and well-being. This concern serves as the principal motivation for our present study. Overall, this study employs a person-centered approach to seek potential latent profiles of attachment through indicators related to attachment to the mother, the father, and peers from an adolescent perspective and to explore if these profiles exhibit variances by variables associated with these indicators.

### Developmental trajectory of attachment during adolescence

*Attachment* is a biologically grounded, stable, and lasting connection established by reciprocal interactions between individuals and significant others. Attachment theory proposes that individuals develop positive or negative internal working models (mental representations) of themselves and others based on their interactions with primary caregivers during their early years (Bowlby, 1969, 1973, 1988). The internal working models guide individuals’ cognition, emotions, and behaviors during their lifespan and significantly affect various constructs, including personality development, identity formation, and interpersonal relationships (Bretherton and Munholland, 2008; Main et al., 1985). The models are reinforced through a developmental process, ultimately attaining their definitive form during adolescence and becoming more resistant to change (Zimmermann and Becker-Stoll, 2002).

The developmental trajectory of attachment during adolescence progresses from a state of dependence on parents to a state of autonomy (Moretti et al., 2018; Steinberg, 2007; Weiss, 1991). The growing competencies diminish reliance on parents while enhancing the aspiration for autonomy and the pursuit of novel experiences (Allen and Tan, 2016). These normative changes, which are essential for healthy development and fostering autonomy (Branje, 2018), may prompt teenagers to renegotiate their attachment to their parents (Allen and Tan, 2016; McElhaney et al., 2009). As teenagers strive to transition from the typical reliance of childhood to the expected independence of adulthood, parents concurrently encounter novel methods of supporting their children within a relational framework that diverges from earlier developmental phases (Moretti and Peled, 2004). While it is anticipated that the attributes of parent attachment during individuation will evolve to foster healthy communication, autonomy, openness, and emotional intimacy (Koehn and Kerns, 2018; Steinberg, 2007; Zimmermann and Becker-Stoll, 2002), individual differences may be evident throughout this process. Adolescents with insecure attachment styles (insecure-avoidant, insecure-anxious, insecure-disorganized) may experience a more troublesome process of differentiation and individuation (Delgado et al., 2022). They may view conflicts during this phase as threats, prompting them to evade both confrontations and possibilities for settlement. Conversely, securely attached teenagers can navigate conflicts more effectively by recognizing that these disputes will not jeopardize their relationship with their parents (Allen, 2008; Allen and Land, 1999).

During adolescence, the developmental trajectory of attachment shifts from a focus on parental relationships to a predominant emphasis on peer interactions. The establishment and maintenance of nurturing friendships assume increased importance for adolescents (Inderbitzen, 1994), which, in turn, there is a normative increase in the time spent with peers (Gorrese and Ruggieri, 2012; Hayre et al., 2024; Moretti and Peled, 2004). Increased frequency of peer interactions then paves the way for adolescents to initiate peer attachment outside the family system (Allen, 2008; Armsden and Greenberg, 1987; Gorrese and Ruggieri, 2012). In this process, many adolescents begin to evaluate the possibility of finding a peer group as a source of security and support, while still maintaining parental ties (Kobak et al., 2007). Peer attachment also develops in those perceiving their peers as a source of social and emotional support and intimacy (Laible, 2007; Laible et al., 2000; Wilkinson, 2010). Overall, adolescence can be regarded as a period of transition in which attachment behaviors shift from parents to peers. Adolescents’ attachment experiences with different individuals may have profound implications on their psychosocial adjustment and functioning (Buist et al., 2002).

### Processes tailored by parent and peer attachment

One salient indicator of adjustment to adolescence-specific developmental changes may be attachment to parents (McElhaney et al., 2009). Secure parent attachment is widely recognized as a foundational component of mental health and facilitates psychosocial adjustment in adolescence (Allen and Tan, 2016; Armsden and Greenberg, 1987; Buist et al., 2002; Greenberg et al., 1983; Oldfield et al., 2016; Raja et al., 1992). Indeed, adolescents with a secure attachment to their parents were previously reported to have positive peer relationships and greater social competence (Gorrese and Ruggieri, 2012), higher self-esteem (Wilkinson, 2004), higher levels of well-being (Mónaco et al., 2019), and greater life satisfaction (Nickerson and Nagle, 2004). Nevertheless, insecurely attached adolescents were linked to a multitude of risk factors, including depressive symptoms (Spruit et al., 2020), anxiety symptoms (Kerns and Brumariu, 2014), internalizing and externalizing problems (Badovinac et al., 2021; Madigan et al., 2016), emotion regulation difficulties, increased risk-taking behaviors (Flykt et al., 2021), low sense of belonging, suicidal ideation (Yang et al., 2023), and poor romantic relationship quality (Gorla et al., 2024). Hence, the relevant literature regards secure attachment to parents as a robust indicator of numerous factors associated with healthy development, including the quality of social relationships, effective emotion regulation skills, and positive behavioral adjustment and functioning (Allen et al., 2018; Barone et al., 2021; Berlin et al., 2008; Collins et al., 2002; Grossmann et al., 2006; Pascuzzo et al., 2013; Sroufe, 2005).

Peer attachment is another significant factor in adolescents’ healthy development, well-being, and psychosocial adjustment (Delgado et al., 2022; Gorrese and Ruggieri, 2012). The literature consistently associates secure peer attachment — defined by an affectionate bond comprising elements of trust, commitment, mutual understanding, and reciprocal emotional expression and communication (Armsden and Greenberg, 1987; Laible et al., 2000) — with a variety of positive outcomes, including heightened school engagement, improved prosocial behaviors (Oldfield et al., 2016), enhanced self-esteem (Gorrese and Ruggieri, 2013), strengthened socio-emotional competencies, and a more positive self-image (Laible et al., 2004). In contrast, insecure peer attachment, characterized by sentiments of communication barriers, social alienation, and isolation (Armsden and Greenberg, 1987), is identified as a significant predictor of criminal activities (McElhaney et al., 2006), internalizing symptoms (Gorrese, 2016), psychological distress, and behavioral problems among adolescents. While peer attachment is widely regarded as a significant indicator of psychosocial adjustment, research consistently highlights that adolescents predominantly seek emotional support from their parents rather than their peers when confronted with challenges (Allen et al., 2003; Kerns et al., 2015; Markiewicz et al., 2006). This finding underscores the interaction between peer and parent attachment in promoting healthy development during adolescence (Moretti and Peled, 2004). A comprehensive understanding of the interplay between mother, father, and peer attachment may then become essential for a nuanced exploration of adolescent development.

### Cultural diversity and differences in parent and peer attachment

Attachment theory is grounded in four core hypotheses: universality, normativity, sensitivity, and competence (Mesman et al., 2016). The first one argues that all infants without severe neurophysiological impairments are capable of forming secure attachments with one or more caregivers, while the normativity hypothesis asserts that secure attachment constitutes the dominant pattern within the overall distribution of attachment classifications. The sensitivity hypothesis emphasizes that secure attachment emerges from caregivers’ sensitive, consistent, responsive, and timely reactions to infants’ needs. In turn, the competence hypothesis assumes that secure attachment provides a developmental foundation for a range of positive outcomes across childhood (van Ijzendoorn and Sagi-Schwartz, 2008). While these four assumptions have substantially contributed to identifying the universal aspects of attachment theory, they simultaneously underscore the necessity of considering cultural and contextual influences in attachment relationships (Mesman et al., 2016). Therefore, although the emergence and expression of attachment patterns display certain universal features, attachment relationships also exhibit meaningful variation shaped by cultural values, social norms, and caregiving practices characteristic of individualistic and collectivistic contexts (Pearson and Child, 2007).

Limited empirical evidence on the cultural and contextual dimensions of attachment during adolescence consistently points to both within-culture and cross-cultural differences in adolescents’ attachment styles. For instance, a recent cross-national study with Italian, Spanish, Polish, and Chinese adolescents demonstrated that Italian and Spanish participants reported stronger attachment to their mothers compared to their Chinese and Polish counterparts. Moreover, mothers emerged as the primary attachment figures relative to fathers across all participants (Mancinelli et al., 2021). Another cross-cultural study examined parental attachment among adolescents from China, Italy, and Costa Rica. These countries are characterized by distinct social values and family orientations. The findings demonstrated that Chinese adolescents exhibit lower levels of parental attachment and report stronger attachment to fathers than to mothers compared to their Costa Rican and Italian peers (Li et al., 2014). In the Turkish context, findings from a large-scale, variable-centered study revealed notable regional differences in adolescents’ parental attachment. Adolescents living in Central Anatolia, Eastern Anatolia, and the Black Sea regions reported higher levels of parental attachment compared to those living in Southeastern Anatolia, Marmara, the Mediterranean, and the Aegean regions. These within-culture variations were associated with prevailing family models in Türkiye. The findings highlighted the influential roles of environmental and contextual factors alongside individual characteristics in shaping attachment processes (Doğan, 2016). In this sense, the present study represents the first person-centered examination of adolescents’ parent and peer attachment from their perspectives in a Turkish sample. Given that Turkish culture is characterized by the coexistence of individualistic and collectivistic psycho-cultural elements and is often conceptualized through the notion of a “relational self” (Kagitcibasi, 2005), the study is expected to provide a rich and heterogeneous context for examining attachment patterns. In this respect, the present research constitutes a meaningful and original attempt to elucidate how the universality, normativity, and competence hypotheses are reflected and negotiated in the Turkish context.

### Adolescent outcome indicators in the context of parent and peer attachment

Attachment relationships during adolescence are consistently linked to a range of outcome indicators reflecting adolescents’ psychological adjustment and functioning. In relation to these relationships, *life satisfaction* emerges as a prominent construct in variable-centered research. It is conceptualized as one’s cognitive evaluations of their overall lives and/or specific life domains (Diener, 1984, 1994). From an attachment theory perspective, positive internal working models facilitate more favorable evaluations of one’s life experiences (Bowlby, 1973; Main et al., 1985; Bretherton and Munholland, 2008), while negative models may contribute to diminished life satisfaction (Li et al., 2023). Previous findings further indicated that secure parent and peer attachment during adolescence is associated with higher levels of life satisfaction (Greenberg et al., 1983; Ma and Huebner, 2008).

Beyond life satisfaction, *resilience* represents another key construct associated with attachment during adolescence. Resilience is conceptualized as an outcome emerging from the dynamic interplay between risk and protective factors operating across multiple systems, including the individual, family, school, and community contexts (Gartland et al., 2011; Masten and Cicchetti, 2016). More recently, the conceptualization of resilience has expanded to incorporate the development of resilient mindset and meaning-making processes (Arslan and Wong, 2023; Wong, 2020). Considering that resilience is fundamentally grounded in relational dynamics (Luthar, 2006), that attachment shapes interpersonal functioning (Bowlby, 1969; Bowlby, 1973; Bowlby, 1988), and that secure attachment is consistently associated with higher levels of resilience (Bender and Ingram, 2018; Darling Rasmussen et al., 2019; Zhang et al., 2023), it becomes critical to examine the potential role of adolescents’ attachment relationships in shaping their resilient mindset processes.

Another construct closely linked to parent and peer attachment during adolescence is *basic psychological needs*. According to Self-Determination Theory, human behavior is guided by three innate and universal psychological needs: autonomy, competence, and relatedness (Deci and Ryan, 2000; Ryan and Deci, 2000; Deci and Ryan, 2012). Autonomy refers to the experience of volition and willingness, competence to the experience of effectiveness and mastery, and relatedness to experiences of connection, warmth, and belonging (Vansteenkiste et al., 2020). The theory further assumes that the extent to which these needs are satisfied varies across social–relational contexts and is shaped by one’s experiences in those contexts (Erçelik and Dost-Gözkan, 2022). Given that family and peer relationships constitute adolescents’ most proximal social environments, it is reasonable to suggest that attachment relationships with parents and peers play a decisive role in either satisfying or hindering their basic psychological needs (Gao et al., 2022). Prior research highlighted that adolescents who feel valued, important, and loved by their attachment figures tend to perceive their environments as more supportive, which facilitates the satisfaction of their basic psychological needs (La Guardia, 2008). More specifically, adolescents who establish strong bonds with their parents are more likely to display autonomous functioning and to pursue activities aligned with their personal interests (Kocayoruk, 2012). Those who perceive peers as their primary source of social support tend to position peer relationships as a central context for the fulfillment of basic psychological needs (Parent, 2023).

In addition to these constructs, *relative deprivation* represents another outcome potentially associated with attachment relationships during adolescence. It is defined as one’s or groups’ perceptions of being disadvantaged compared to others, their beliefs that such disadvantage is unjust, and their emotional responses such as sadness, frustration, and anger (Smith et al., 2012; Smith and Pettigrew, 2015). Social comparison processes are considered a core psychological mechanism underlying experiences of relative deprivation (Stiles et al., 2000). Despite the scarcity of research examining attachment and relative deprivation during adolescence, a substantial body of studies indicates that attachment styles have a significant role in shaping one’s social comparison tendencies (Bamford and Halliwell, 2009; Puissant et al., 2011). Adolescence is developmentally marked by heightened sensitivity to social comparison in identity formation and self-evaluation (Erikson, 1968) and in interactions with significant others in the immediate social environment (Wang and Meng, 2024). Thus, the present study is guided by the assumption that securely attached adolescents may be less likely to experience feelings of relative deprivation in the context of social comparisons, whereas those with insecure attachment styles may exhibit heightened sensitivity to such comparisons, perceive themselves as more disadvantaged than their peers, and report higher levels of relative deprivation.

In line with the overall view that adolescents’ parent and peer attachment cannot be reduced to a single indicator, examining attachment in relation to multiple outcome domains may offer a more comprehensive understanding of its multifaceted nature. Accordingly, life satisfaction, resilient mindset, basic psychological needs, and relative deprivation are conceptualized as the key psychological outcome indicators in the present study.

### Current study

A considerable body of research on adolescent attachment to the mother, the father, and peers has adopted a variable-centered approach. While variable-centered approaches offer valuable insights, their focus on the independent associations of different dimensions of attachment with specific outcomes may, by default, ignore the potential for significant variability in attachment patterns across individuals. By way of contrast, person-centered approaches are predicated on the principle of organizing the population into mutually exclusive and inclusive subgroups of similar individuals, as opposed to the measurement of a specific variable (Lanza and Cooper, 2016; Lanza and Rhoades, 2013). Considering the multifaceted nature of adolescent attachment and the mounting imperative for research focusing on a range of secure bases and havens in adolescents’ lives (Deneault et al., 2024), it may be advantageous to adopt a person-centered approach when exploring parent and peer attachment from their viewpoints. In fact, a few studies in recent years have yielded valuable insights into the significance of a person-centered approach when examining the potential effects of attachment on psychological outcomes during adolescence (Flykt et al., 2021; Gao et al., 2024; He et al., 2018; Yang and McGinley, 2022). For example, He et al. (2018) identified four distinct profiles of parent and peer attachment in a sample of 941 Chinese adolescents, while Flykt et al. (2021) discovered five distinct profiles of attachment (the mother, the father, close friends, and romantic partners) in a sample of 449 Finnish adolescents. Gao et al. (2024), on the other hand, identified three disparate profiles concerning peer attachment and parent–child relationships across two distinct time periods in a sample of 453 Chinese adolescents. Relying on the contextual boundaries mandated by samples from Eastern and Western cultures, these studies indicate that parent and peer attachment are not always concomitant in adolescence and that different cultures may produce diverse attachment profiles. They also emphasize that different attachment profiles can be associated with various psychological outcomes in adolescence. It is, undoubtedly, imperative to expand the existing limited knowledge of adolescent attachment profiles explored through a person-centered approach by focusing on the data from highly representative, cross-cultural samples. Given the methodological limitations inherent to variable-centered approaches, we adopt a person-centered approach to examine parent and peer attachment in the present study. In this sense, the current research is built on two principal objectives. While the first is to explore the latent profiles/subgroups of parent and peer attachment in a sample of Turkish adolescents aged 14–18 years, the second is to examine if adolescents in these subgroups/profiles vary by certain outcome indicators, such as life satisfaction, resilient mindset, relative deprivation, and basic psychological needs satisfaction and frustration.

## Method

### Participants and procedure

The target population of this cross-sectional study consisted of a sample of 306.225 students aged 14–18 years who were enrolled in public and private high schools offering general, religious, and vocational-technical secondary education in Ankara during the 2023–2024 academic year (MoNE, 2023). We recruited participants for the study using multistage cluster sampling to ensure the probabilistic and generalizable nature of the research. Accordingly, we included only adolescents attending 9th-12th grades at public/private high schools affiliated with the Ministry of National Education (MoNE) in Ankara in the 2023–2024 academic year, being aged 14–18 years, and providing both parental and personal consent for participation in the study. Moreover, we sought approval from the school administration for their participation, as well as necessary bureaucratic permissions from the MoNE for the study. Nevertheless, the following criteria were considered reasons for exclusion: the decision to withdraw from the study, the absence of informed consent forms, the incompleteness of at least 20% of data collection tools, the diagnosis of any special needs, and the single-parent family structure (deceased mother or father).

The Social and Human Sciences Research Ethics Committee of Hacettepe University (Approval No: E-66777842-300-00003284857, dated January 2, 2024) and the Ankara Provincial Directorate of National Education granted ethical approval and permission for implementation, respectively (Approval No: E-14588481-605.99-96708060, dated February 14, 2024). All procedures carried out in this study involving human participants were by the 1964 Helsinki Declaration and its subsequent amendments or equivalent ethical standards. As the research does not fall under the scope of a clinical trial, no trial registration was required. With the necessary ethical permissions in place, we initiated the data collection process, beginning with sample selection. In the first stage of multistage cluster sampling, we generated the most up-to-date list of public and private secondary schools in the Ankara province and selected 38 of them using a systematic selection technique (proportional to size). The administrators of these selected schools were informed about the content and scope of the study via telephone and e-mail and through face-to-face meetings, when necessary, all selected schools provided permission for implementation. In the second stage, we randomly selected a class from each grade level (9th to 12th grades) in the schools that agreed to participate, resulting in a total of 144 classes from 36 schools. Given that the average class size in schools in Ankara is about 25 students, we expected to invite approximately 3,600 students to participate in the study. Overall, the final number of participants was expected to amount to about 2,880, even in the case that 20% of students might decline to participate in the research or fail to respond to the survey questions.

We informed a total of 2,961 students from these 36 schools about the principal objectives and projected duration of the research, the protocol for data confidentiality, the principle of voluntary participation, and their right to withdraw from the study at any point in time. We informed the participants that their personal information would remain confidential under all circumstances and that the study’s findings might be published. Since the manuscript does not contain identifiable personal data (such as names, images, or other individual details), we did not obtain specific consent for publication. In collaboration with the school administrators, we collected the data from voluntary students at a suitable time. The research data were collected from February 22 to May 21, 2024. Prior to participation, all student volunteers were thoroughly informed about the study and provided written informed assent. Additionally, parental informed consent forms were sent to the parents via the student participants, who were also clearly informed about the submission deadline for these forms as well as the scheduled date of data collection.

During the procedure, 160 students opted out of participating in the study, while 52 withdrew midway through the research. Moreover, we excluded 50 with a documented diagnosis of special needs and 54 reporting a single-parent family structure. The final data set consisted of responses from 2,645 students; however, the data from 113 participants who left more than 20% of the scale items unanswered and responses from 210 participants identified as outliers were excluded from the analyses, resulting in a final sample of 2,322 high school students (*M*_age_ = 15.91, SD = 1.13; females = 57.1%). The distribution of participants by grade level was as follows: 29.8% in 9th grade, 26.8% in 10th grade, 25.5% in 11th grade, and 17.9% in 12th grade.

### Data collection tools

Inventory of Parent and Peer Attachment (IPPA): Armsden and Greenberg (1987) designed this inventory to assess the adolescent’s level of attachment to their parents and peers. The IPPA was adapted into Turkish by Kocayörük (2010). Although the original inventory comprises 25 items for each scale, the adaptation study rearranged each scale to 18 items. The inventory is divided into three parts. While Part I prompts the adolescent to evaluate their attachment experiences with their mother, Part II asks the adolescent to reflect on their experiences with their father. Part III focuses on the adolescent’s relationships with a close friend. These three parts address attachment-related experiences through three sub-scales operating within the theoretical framework of attachment theory: trust, communication, and alienation. The trust sub-scale assesses the level of trust the adolescent feels toward their parents/peers, the mutual understanding and respect, and the extent to which they feel their parents/peers trust them in the attachment relationship (e.g., “I feel my mother does a good job as my mother.”, “I wish I had a different father.”, “I can count on my friends when I need to get something off my chest.”). The communication subscale assesses the adolescent’s perceptions of how sensitive and responsive parents/peers are to their emotional states and concerns, and the scope and quality of verbal communication in their attachment relationships (e.g., “If my mother knows something is bothering me, she asks me about it.” “I tell my father about my problems and troubles.”, “My friends care about how I am feeling.”). Finally, the alienation subscale assesses the degree to which the adolescent experiences feelings of isolation, anger, and a sense of alienation from their parents/peers in their attachment relationships (e.g., “I get upset a lot more than my mother knows about.”, “My father doesn’t understand what I’m going through these days.”, “It seems as if my friends are irritated with me for no reason.”). The items on the inventory are rated on a 5-point Likert-type scale ranging from 1 (never true) to 5 (always true). High scores on the trust and communication subscales and low scores on the alienation subscale are indicative of secure attachment.

The measurements from the current sample produced a good model-data fit for Part I (mother attachment scale) with the following indices: Root Mean Square Error of Approximation (RMSEA) = 0.059 (90% CI = 0.056–0.062), Standardized Root Mean Squared Residual (SRMR) = 0.042, Comparative Fit Index (CFI) = 0.993, Tucker-Lewis Index (TLI) = 0.992, Normed Fit Index (NFI) = 0.992, and Goodness-of-Fit Index (GFI) = 0.995. For reliability concerns, we calculated Cronbach’s alpha (*α*) and McDonald’s omega (*ω*) coefficients to be 0.622 and 0.650 for the trust subscale, 0.942 and 0.943 for the communication subscale, 0.671 and 0.684 for the alienation subscale, and 0.931 and 0.936 for the total Part I score, respectively. The confirmatory factor analysis (CFA) also yielded a good model-data fit for Part II (father attachment scale) with the following indices RMSEA = 0.028 (90% CI = 0.024–0.031), SRMR = 0.031, CFI = 0.997, TLI = 0.997, NFI = 0.996, and GFI = 0.997. Cronbach’s α and McDonald’s ω were 0.788 and 0.803 for the trust subscale, 0.954 and 0.954 for the communication subscale, 0.712 and 0.716 for the alienation subscale, and 0.940 and 0.943 for the total Part II score, respectively. Part III (peer attachment scale) also demonstrated a good model-data fit: RMSEA = 0.041 (90% CI = 0.037–0.044), SRMR = 0.044, CFI = 0.986, TLI = 0.984, NFI = 0.983, and GFI = 0.990. Cronbach’s *α* and McDonald’s *ω* were 0.852 and 0.859 for the trust subscale, 0.822 and 0.824 for the communication subscale, 0.773 and 0.778 for the alienation subscale, and 0.898 and 0.897 for the total Part III score, respectively.

Satisfaction with Life Scale (SWLS): Developed by Diener et al. (1985) to assess one’s life satisfaction, the SLWS was adapted into Turkish by Yetim (1993). The single-factor scale consists of five items (e.g., “I am satisfied with my life”), and the participant responses are scored on a 7-point scale ranging from 1 (strongly disagree) to 7 (strongly agree). In this study, the CFA produced the following fit indices RMSEA = 0.023 (90% CI = 0.004–0.042), SRMR = 0.019, CFI = 0.999, TLI = 0.998, NFI = 0.998, and GFI = 0.999. In addition, we calculated Cronbach’s α and McDonald’s ω to be 0.830 and 0.830 for the total SLWS score, respectively.

Resilient Mindset Scale (RMS): The RMS was designed by Arslan and Wong (2023) to assess the adolescent’s level of resilient mindset. The scale comprises six items (e.g., “I have the mental and emotional toughness to withstand the challenges that I face or come my way.”). Participant responses to the RMS items are scored on a 5-point Likert-type scale ranging from 0 (almost never true) to 4 (almost always true). Higher scores on the scale indicate a higher level of resilient mindset. The measurements from the current sample produced a good model-data fit for the RMS with the following indices: RMSEA = 0.035 (90% CI = 0.023–0.048), SRMR = 0.029, CFI = 0.994, TLI = 0.991, NFI = 0.993, and GFI = 0.998. In addition, we calculated Cronbach’s *α* and McDonald’s *ω* to be 0.800 and 0.802 for the total RMS score, respectively.

Basic Psychological Need Satisfaction and Frustration Scale (BPNSFS): Chen et al. (2015) developed the BPNSFS to measure one’s perceived psychological needs satisfaction and frustration, and Mouratidis et al. (2018) adapted it into Turkish. The scale consists of 24 items within two components: basic psychological needs satisfaction (BPNS) and basic psychological needs frustration (BPNF). Each of these components has three subscales. While the BPNS component covers items related to autonomy satisfaction (e.g., “I feel I have been doing what really interests me.”), relatedness satisfaction (e.g., “I experience a warm feeling with the people I spend time with.”), and competence satisfaction (e.g., “I feel I can successfully complete difficult tasks.”), the BPNF component includes items related to autonomy frustration (e.g., “My daily activities feel like a chain of obligations.”), relatedness frustration (e.g., “I feel the relationships I have are just superficial.”), and competence frustration (e.g., “I feel like a failure because of the mistakes I make.”). Scores on the scale range from 1 (completely disagree) to 5 (completely agree); high scores indicate either higher need satisfaction or frustration.

In this study, the CFA yielded the following fit indices for the total BPNSFS score: RMSEA = 0.038 (90% CI = 0.035–0.040), SRMR = 0.042, CFI = 0.980, TLI = 0.976, NFI = 0.974, and GFI = 0.988. Moreover, we calculated Cronbach’s α and McDonald’s ω to be 0.749 and 0.752 for autonomy satisfaction, 0.792 and 0.793 for autonomy frustration, 0.644 and 0.648 for relatedness satisfaction, 0.778 and 0.783 for relatedness frustration, 0.849 and 0.849 for competence satisfaction, 0.793 and 0.796 for competence frustration, 0.801 and 0.791 for the BPNS component, and 0.855 and 0.856 for the BPNF component, respectively.

Relative Deprivation Scale-Adolescent Form (RDS-AF): Mucuk and Sahin (2023) designed the scale to assess the relative deprivation levels of Turkish adolescents within the contexts of school (e.g., “I feel like the school administration bestows privileges on my friends”), family (e.g., “I feel like my family does not spend enough time with me when I compare myself to my peers”), and economic (e.g., “I am not happy when I compare my financial possibilities with my peers”). Sixteen items on the scale are scored on a scale ranging from 1 (strongly disagree) to 7 (strongly agree), and higher scores suggest a higher level of relative deprivation. In the present study, we concluded a good model-data fit for the total RDS-AF score with the following fit indices: RMSEA = 0.033 (90% CI = 0.029–0.037), SRMR = 0.042, CFI = 0.989, TLI = 0.987, NFI = 0.985, and GFI = 0.992. We also computed Cronbach’s α and McDonald’s ω to be 0.849 and 0.846 for the school subscale, 0.873 and 0.874 for the family subscale, 0.830 and 0.833 for the economic subscale, and 0.884 and 0.879 for the total score, respectively.

### Data analysis

The research data were analyzed using IBM SPSS, JASP, and Mplus. We considered skewness and kurtosis statistics to evaluate the normality of the data distribution. Data with skewness values up to ±3.00 and kurtosis values up to ±10.0 suggest a close proximity of the dataset to a normal distribution (Kline, 2015). Moreover, we computed *z*-scores for each variable to detect univariate outliers (Yan and Su, 2009). The construct validity of the measurement tools was sought through CFA where we selected the Diagonally Weighted Least Squares (DWLS) method as the parameter estimation technique and considered an array of goodness-of-fit indices, including RMSEA, SRMR, NFI, TLI, GFI, and CFI (Gana and Broc, 2019). For reliability concerns, we also calculated Cronbach’s alpha and McDonald’s omega coefficients for the measurements from the present sample. While a reliability coefficient between 0.60 and 0.70 is considered acceptable reliability (van Griethuijsen et al., 2015), a coefficient of 0.70 and above is regarded as high reliability (Salvucci et al., 1997). Initially, we ran Pearson’s correlation analysis to uncover the relations between the research variables. Next, we utilized the latent profile analysis (LPA) to reveal the latent profiles of participants’ parent and peer attachment. LPA is capable of generating latent subpopulations within the target population based on a specific variable set. In the present study, we identified a number of attachment profiles using several methods, including the Akaike Information Criterion (AIC), Bayesian Information Criterion (BIC), Sample-Adjusted Bayesian Information Criterion (SABIC), Entropy Value, Vuong-Lo–Mendell–Rubin Likelihood Ratio Test (VLMR LRT), Lo–Mendell–Rubin Adjusted Likelihood Ratio Test (LMR-A LRT), and Bootstrap Likelihood Ratio Test (BLRT). In LPA, we initially estimated a one-profile solution, and the number of profiles was then gradually increased as the analysis progressed. Upon deciding an optimal number of profiles based on the fit values, we terminated the procedure and labeled the identified latent profiles (Spurk et al., 2020). Finally, a Welch’s ANOVA was conducted to compare participants’ scores on the research variables (life satisfaction, resilient mindset, relative deprivation, and BPNS/F) across the identified attachment profiles, given that the normality assumption was met while the homogeneity of variances assumption was not. Pairwise comparisons were then made using the Games-Howell Test due to the non-homogeneity of the variances. Effect sizes were reported as eta squared (η^2^).

## Results

### Common method bias

The utilization of a unidimensional survey form, incorporating self-report instruments, may introduce common method bias. To minimize this bias and the order effect and maximize the reliability and validity of the results, the data collection instruments were made anonymous and administered to participants in a randomized order. Moreover, we explored the likelihood of common method bias using Harman’s single-factor test. The test yielded 17 factors with eigenvalues greater than one, with 20.764% of the variance being explained, in the instruments administered to participants. As this rate falls below the critical threshold of 40%, we may claim that our findings were not significantly biased by the research method adopted in this study (Podsakoff et al., 2003).

### Descriptive statistics and correlation analysis

We considered participating adolescents’ IPPA subscale scores in LPA to uncover the latent profiles/subgroups of their parent and peer attachment. Prior to the respective analysis, we explored the descriptive statistics and relations of the variables. The initial findings are presented in Table 1.

**Table 1:** Descriptive statistics and relationships of parent/peer attachment inventories (*n* = 2,322).

Variables	Min.	Max.	$\bar{X}$	SD	Skewness	Kurtosis	1	2	3	4	5	6	7	8
1. Mother trust	27.49	56.09	51.68	6.89	−1.74	2.38	--							
2. Mother communication	24.75	61.80	51.05	9.08	−0.68	−0.37	0.67^**^	--						
3. Mother alienation	35.64	75.66	49.20	9.52	0.42	−0.64	−0.42^**^	−0.59^**^	--					
4. Father trust	23.06	57.54	51.30	8.38	−1.43	1.18	0.33^**^	0.27^**^	−0.27^**^	--				
5. Father communication	32.21	65.49	50.85	9.56	−0.21	−0.97	0.29^**^	0.45^**^	−0.37^**^	0.69^**^	--			
6. Father alienation	33.24	69.60	49.46	9.81	0.24	−0.82	−0.22^**^	−0.34^**^	0.60^**^	−0.36^**^	−0.52^**^	--		
7. Peer trust	20.20	63.15	50.63	9.30	−0.66	−0.25	0.15^**^	0.16^**^	−0.11^**^	0.13^**^	0.15^**^	−0.09^**^	--	
8. Peer communication	24.02	64.14	50.70	9.20	−0.49	−0.30	0.16^**^	0.21^**^	−0.18^**^	0.14^**^	0.20^**^	−0.16^**^	0.74^**^	--
9. Peer alienation	35.61	79.29	49.39	9.56	0.62	−0.21	−0.20^**^	−0.22^**^	0.41^**^	−0.20^**^	−0.21^**^	0.42^**^	−0.45^**^	−0.50^**^

**p < 0.01.

The skewness and kurtosis statistics suggested that participants’ IPPA scores showed an almost normal distribution. *T*-scores also indicated that adolescents held similar perceptions of their parents and peers. In the correlation analysis, no variable pair showed an association greater than 0.80, indicating the absence of multicollinearity issues.

### Latent profiles of parent and peer attachment

One to 10 LPA models were tested and comparatively analyzed regarding goodness-of-fit indices to discover the latent profiles of participating adolescents’ attachment to mother, father, and peers (trust, communication, alienation; Table 2).

**Table 2:** Summary of the different class solutions obtained from the latent profile analysis (LPA).

Number of classes	Number of parameters	AIC	BIC	SABIC	*p-*values			Entropy	Class proportions	Smallest profile (*n*)
					VLMR LRT	LMR-A LRT	BLRT			
1	18	151,116.12	151,219.63	151,162.44						
2	28	147,209.67	147,370.68	147,281.72	0.000	0.000	0.000	0.815	0.38458/0.61542	893
3	38	145,589.68	145,808.19	145,687.45	0.000	0.000	0.000	0.838	0.37726/0.47115/0.15159	352
4	48	144,588.10	144,864.11	144,711.60	0.002	0.002	0.000	0.860	0.30017/0.44358/0.11456/0.14169	266
5	58	143,760.28	144,093.80	143,909.52	0.000	0.000	0.000	0.878	0.08053/0.11240/0.43066/0.28854/0.08786	187
6	68	143,097.84	143,488.85	143,272.80	0.003	0.003	0.000	0.862	0.08355/0.08096/0.14341/0.19638/0.10680/0.38889	188
**7**	**78**	**142,692.10**	**143,140.61**	**142,892.79**	**0.009**	**0.009**	**0.000**	**0.850**	**0.10680/0.07967/0.07321/0.10767/0.08269/0.33075/0.21921**	**170**
8	88	142,274.67	142,780.69	142,501.10	0.311	0.316	0.000	0.883	0.02929/0.05168/0.08915/0.11197/0.12748/0.04823/0.37468/0.16753	68
9	98	141,929.03	142,492.55	142,181.19	0.678	0.679	0.000	0.871	0.04953/0.02842/0.04177/0.11413/0.10250/0.09302/0.18131/0.32687/0.06245	66
10	108	141,676.56	142,297.58	141,954.44	0.664	0.665	0.000	0.881	0.04910/0.03359/0.05297/0.34238/0.09991/0.04608/0.02929/0.16968/0.11154/0.06546	68

Bold values indicate the number of parameters, information criteria, entropy, model comparison tests, class proportions, and the smallest class size for the selected seven-class solution.

**Figure 1:**
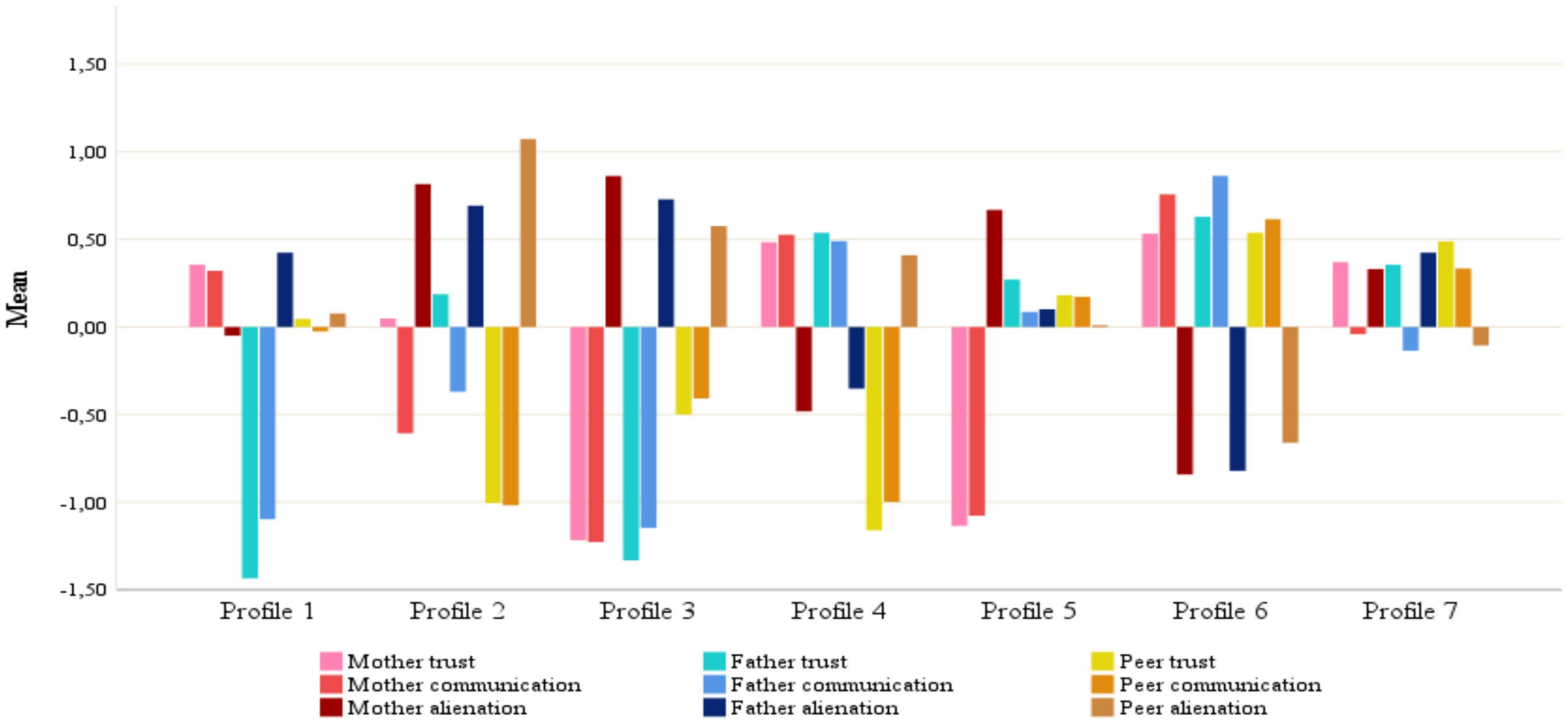
Standardized means for indicators of maternal, paternal and peer attachment for the seven-profile solution. Profile 1: predominantly insecure attachment to the father; Profile 2: high parental distance and insecure peer attachment; Profile 3: insecure attachment to mother, father, and peers; Profile 4: secure attachment to mother and father but insecure attachment to peers; Profile 5: predominantly insecure attachment to the mother; Profile 6: secure attachment to mother, father and peers; Profile 7: low parental distance and secure peer attachment.

The values of information criteria (AIC, BIC, and SABIC) concomitantly decreased as the number of profiles increased. The entropy value exceeded 0.80 for all profiles and demonstrated an upward trend as the number of profiles increased. The *p*-value of the BLRT statistic was significant for all profiles; however, it was not the case for the *p*-values from the VLMR LRT and LMR-A LRT statistics for the eighth and subsequent profiles. Overall, we decided to analyze the seven-profile model in the present study. Figure 1 presents *z*-scores for the nine attachment indicators for this seven-profile model.

The indicators of parent and peer attachment across these seven distinct profiles varied considerably. Therefore, we considered the predominant attributes in each profile when designating the profiles. Participants in Profile 1 (*n* = 248, 10.7%) demonstrated above-average trust and communication and below-average alienation with their mothers; significantly low trust and communication and high alienation with their fathers; and average peer trust, communication, and alienation. As this profile is characterized by a lack of trust, limited communication, and a distant relationship—particularly with the father—we designated this profile as the “Predominantly Insecure Attachment to the Father.”

Adolescents in Profile 2 (*n* = 145, 8%) exhibited average trust, low communication, and high alienation with their mothers; moderately high trust, low communication, and high alienation with their fathers; and poor peer trust and communication, and significantly high peer alienation. It is noteworthy that although adolescents in this profile reported an average trust in their parents, their experiences indicated high communication challenges in their relationships with their parents, resulting in a sense of alienation. This profile is, thus, characterized by an absence of trust, inadequate communication, and a pervasive sense of alienation, particularly in the context of peer relationships. Consequently, it was designated as “High Parental Distance and Insecure Peer Attachment.”

Participants in Profile 3 (*n* = 170, 7.3%) displayed poor trust and communication and high alienation in their relationships with their parents. In addition, they had low peer trust and communication with high peer alienation. As this profile seems to be characterized by a pervasive lack of trust, communication, and alienation in the relationships with parents and peers, we designated it as “Insecure Attachment to Mother, Father, and Peers.”

In Profile 4 (*n* = 250, 10.8%), we discovered high trust and communication with low alienation in participants’ relationships with their parents. Conversely, they tended to exhibit low peer trust in and communication and pronounced peer alienation. Therefore, this profile was designated “Secure Attachment to Mother and Father but Insecure Attachment to Peers.”

Adolescents in Profile 5 (n = 192, 8.3%) had low trust and communication and high alienation in their relationships with their mothers. They also exhibited slightly above-average trust, communication, and alienation in their relationships with their fathers and peers. This profile is thus typified by a pronounced absence of trust, communication, and intimacy in adolescent-mother relationships, leading us to refer it to as “Predominantly Insecure Attachment to the Mother.”

Profile 6 (*n* = 768, 33.1%) appeared with elevated trust and communication, as well as low alienation, among adolescents toward their parents and peers. This profile was thus designated “Secure Attachment to Mother, Father, and Peers.”

Adolescents in Profile 7 (*n* = 509, 21.9%) exhibited above-average trust but below-average communication in their relationships with their parents. In contrast, they had above-average trust and communication and below-average alienation in their peer relationships. Although participants reported feelings of security to their parents, this profile can be construed as a noticeable absence of communication and the presence of emotional distance in adolescent-parent relationships. Hence, this profile was designated as “Low Parental Distance and Secure Peer Attachment.”

### Research variables by latent profiles of parent and peer attachment

This study employed Welch’s ANOVA to explore if participating adolescents’ life satisfaction, resilient mindset, BPNS/F (autonomy, relatedness, competence), and relative deprivation (school, family, and economic) differ by the latent profiles discovered (Table 3).

**Table 3:** Welch’s ANOVA findings regarding profile differences according to the outcome indicators of the research.

Variables	Profile1^(1)^	Profile2^(2)^	Profile3^(3)^	Profile4^(4)^	Profile5^(5)^	Profile6^(6)^	Profile7^(7)^	𝐹_𝑤_	η2	Levene	Games-Howell Test
	*M* (SD)	*M* (SD)	*M* (SD)	*M* (SD)	*M* (SD)	*M* (SD)	*M* (SD)				
Life satisfaction	17.90 (6.33)	16.59 (5.34)	13.95 (5.32)	21.58 (6.13)	17.40 (6.25)	24.47 (5.73)	19.38 (6.08)	138.95^***^	0.248	3.42^**^	(6 > 1;2;3;4;5;7), (4 > 1;2;3;5;7), (7 > 1;2;3;5), (1;2;5 > 3)
Resilient mindset	14.48 (4.79)	13.09 (4.95)	12.84 (4.99)	16.31 (4.20)	13.55 (5.10)	17.36 (4.24)	15.37 (4.72)	47.03^***^	0.111	4.45^***^	(6 > 1;2;3;4;5;7), (4 > 1;2;3;5), (7 > 2;3;5), (1 > 3)
Basic psychological need satisfaction	43.24 (7.64)	38.43 (6.96)	38.06 (7.45)	42.88 (7.41)	41.57 (7.26)	48.71 (6.39)	44.25 (6.45)	107.90^***^	0.217	3.52^**^	(6 > 1;2;3;4;5;7), (7 > 2;3;5), (4 > 2;3), (5;1 > 2;3)
Autonomy satisfaction	14.22 (3.35)	12.68 (3.13)	12.44 (3.48)	14.82 (3.21)	13.50 (3.28)	16.46 (2.74)	14.59 (3.05)	78.80^***^	0.166	5.42^***^	(6 > 1;2;3;4;5;7), (4;7 > 2;3;5), (1 > 2;3), (5 > 3)
Relatedness satisfaction	15.54 (3.23)	13.39 (3.03)	13.69 (3.57)	13.65 (3.32)	15.02 (3.04)	16.38 (2.96)	15.81 (2.71)	48.00^***^	0.118	4.23^***^	(6 > 1;2;3;4;5;7), (7 > 2;3;4;5), (5;1 > 2;3;4)
Competence satisfaction	13.47 (3.94)	12.36 (3.96)	11.94 (4.17)	14.41 (3.81)	13.06 (3.97)	15.87 (3.33)	13.85 (3.72)	50.65^***^	0.114	3.93^***^	(6 > 1;2;3;4;5;7), (4 > 2;3;5), (7 > 2;3), (1 > 3)
Basic psychological need frustration	33.72 (10.00)	38.51 (8.70)	38.07 (9.03)	33.23 (8.98)	34.05 (8.70)	25.38 (8.11)	32.78 (9.04)	114.12^***^	0.216	4.02^***^	(1;2;3;4;5;7 > 6), (2;3 > 1;4;5;7)
Autonomy frustration	12.39 (4.45)	13.68 (3.78)	13.72 (4.07)	11.49 (3.98)	12.64 (3.78)	9.45 (3.75)	12.52 (3.98)	66.87^***^	0.142	3.25^**^	(1;2;3;4;5;7 > 6), (2;3;5;7 > 4), (2;3 > 1;7)
Relatedness frustration	9.44 (3.94)	11.92 (3.90)	11.17 (4.08)	10.56 (3.79)	9.48 (3.93)	7.05 (3.34)	8.68 (3.72)	74.98^***^	0.161	6.14^***^	(1;2;3;4;5;7 > 6), (2;3;4 > 1;7), (2;3 > 5), (2 > 4)
Competence frustration	11.89 (4.48)	12.90 (3.79)	13.18 (3.98)	11.18 (4.06)	11.92 (3.86)	8.88 (3.64)	11.58 (4.06)	63.49^***^	0.132	5.58^***^	(1;2;3;4;5;7 > 6), (3> 1;4;5;7), (2 > 4;7)
Relative deprivation	46.35 (20.50)	47.58 (18.65)	55.00 (19.13)	35.66 (16.57)	47.57 (17.08)	28.42 (12.48)	40.51 (16.30)	118.25^***^	0.223	26.37^***^	(1;2;3;4;5;7 > 6), (1;2;3;5;7 > 4), (1;2;3;5 > 7), (3 > 1;2;5)
School	16.54 (9.50)	16.86 (9.01)	16.64 (8.86)	16.47 (9.29)	17.08 (8.27)	13.35 (8.14)	15.79 (8.55)	12.02^***^	0.029	3.57^**^	(1;2;3;4;5;7 > 6)
Family	17.01 (8.66)	17.83 (8.44)	23.04 (8.59)	9.92 (6.29)	18.21 (7.98)	7.95 (4.65)	14.15 (7.09)	193.91^***^	0.329	60.20^***^	(1;2;3;4;5;7 > 6), (1;2;3;5;7 > 4), (1;2;3,5 > 7), (3 > 1;2;5)
Economic	12.80 (7.88)	12.88 (7.11)	15.32 (7.94)	9.26 (6.04)	12.28 (6.92)	7.12 (3.84)	10.57 (6.08)	79.42^***^	0.159	60.33^***^	(1;2;3;4;5;7 > 6), (1;2;3;5 > 4;7), (3 > 1;2;5)

**p < 0.01; ***p < 0.001. Profile 1: predominantly insecure attachment to the father; Profile 2: high parental distance and insecure peer attachment; Profile 3: insecure attachment to mother, father, and peers; Profile 4: secure attachment to mother and father but insecure attachment to peers; Profile 5: predominantly insecure attachment to the mother; Profile 6: secure attachment to mother, father and peers; Profile 7: low parental distance and secure peer attachment. Numbers (1)–(7) correspond to the seven latent profiles (Profile1–Profile7) and are used as shorthand labels in pairwise comparisons. For example, (6 > 1;2;3;4;5;7) indicates that Profile 6 is higher than Profiles 1, 2, 3, 4, 5, and 7.

The analysis revealed that adolescents’ life satisfaction significantly differed by attachment profiles. In pairwise comparisons, adolescents in Profile 3 exhibited significantly poorer life satisfaction than their counterparts in the other profiles. However, adolescents in Profile 6 had higher levels of life satisfaction than those in the other profiles. Profile 6 was followed by Profile 4; adolescents in Profile 4 exhibited higher levels of life satisfaction than participants in the other profiles, except for Profile 6. Finally, adolescents in Profile 7 had higher life satisfaction scores compared to those in Profiles 1, 2, 3, and 5. Therefore, it can be proposed that secure attachment to parents and peers during adolescence may be an important psychosocial factor supporting life satisfaction.

There were also significant differences between participants’ resilient mindset scores by attachment profiles. Pairwise comparisons showed that adolescents in Profile 6 had significantly higher resilient mindset scores than those in the other profiles. Profile 6 was followed by Profile 4; the resilient mindset scores of adolescents in Profile 4 were significantly higher than those in Profiles 1, 2, 3, and 5. Similarly, participants in Profile 7 had significantly higher resilient mindset scores compared to those in Profiles 2, 3 and 5. The respective scores of adolescents in Profile 1 were also significantly higher than those in Profile 3. Secure attachment can be considered an important psychosocial factor in terms of resilient mindset patterns during adolescence.

Regarding the BPNS, we found significant differences between participants’ total BPNS and subscale scores (autonomy satisfaction, relatedness satisfaction, and competence satisfaction) by attachment profiles. Pairwise comparisons yielded overlapping patterns of results for the total BPNS and subscale scores to a certain degree. For example, adolescents in Profile 6 attained significantly higher BPNS (total BPNS and subscale scores) than those in the other profiles, suggesting that adolescents with secure parent and peer attachment tend to exhibit increased autonomy in decision-making, an enhanced sense of self-efficacy, and a capacity to establish more robust relationships with people around than those in the other attachment profiles. In terms of the total BPNS score, Profile 6 was followed by Profile 7; adolescents in Profile 7 had significantly higher scores compared to Profiles 2, 3, and 5. Similarly, participants in Profiles 1, 4, and 5 had significantly higher total BPNS scores than those in Profiles 2 and 3.

Given participants’ autonomy satisfaction scores, Profile 6 was followed by Profiles 4 and 7. Pairwise comparisons yielded that adolescents in both profiles had significantly higher autonomy satisfaction scores than those in Profiles 2, 3, and 5. Participants in Profile 1 scored significantly higher on the autonomy satisfaction subscale than participants in Profiles 2 and 3; this was also the case between adolescents in Profile 5 and Profile 3. Yet, there was no significant difference between adolescents in Profile 3 (lowest scores) and Profile 2 by autonomy satisfaction. The findings, therefore, suggest that the satisfaction of autonomy needs during adolescence can be supported not by a single attachment figure but secure attachment relationships with both parents and peers.

Adolescents in Profile 6 hit the highest relatedness satisfaction scores. We found participants in Profile 7 to score significantly higher on the respective subscale than those in Profiles 2, 3, 4, and 5. Moreover, the relatedness satisfaction scores of adolescents in Profiles 1 and 5 were significantly higher than those of participants in Profiles 2, 3, and 4. In addition, the analysis revealed that adolescents in Profile 2 exhibited the lowest levels of relatedness need satisfaction. This profile was followed by Profile 3, and we found no significant difference between these profiles by relatedness satisfaction. Thus, our findings suggest that secure attachment to peers during adolescence may play a relatively more prominent role in the satisfaction of relatedness needs.

Apart from participants in Profile 6, who scored the highest on the competence satisfaction subscale, we found adolescents in Profile 4 to have significantly higher competence satisfaction scores than those in Profiles 2, 3, and 5. Similarly, participants in Profile 7 had significantly higher competence satisfaction scores than those in Profiles 2 and 3. This was also the case between adolescents in Profile 1 and those in Profile 3. Upon these findings, we may assert that secure parent and peer secure attachment may assume a key protective role in promoting the satisfaction of autonomy, relatedness, and competence satisfaction in adolescents.

In regard to BPNF, we found significant differences between adolescents’ total BPNF and subscale scores (autonomy frustration, relatedness frustration, and competence frustration) by attachment profiles. Our findings demonstrated that adolescents in Profile 6 attained significantly the lowest BPNF compared to the other profiles, suggesting that adolescents with secure parent and peer attachment may possess a heightened sense of autonomy, self-efficacy, and a reduced risk of social isolation. Yet, participants in Profile 2 had the highest BNPF scores, followed by those in Profile 3. Although the score differences between adolescents in Profiles 2 and 3 were not statistically significant, their scores significantly differed from those in the other profiles. Overall, the results indicate that both a pattern of insecure attachment to parents and peers and a relational configuration characterized by heightened communication problems with parents with insecure peer attachment constitute relational dynamics that hinder the satisfaction of basic psychological needs during adolescence.

In terms of autonomy frustration scores, Profile 6 was followed by Profile 4; adolescents in this profile seem to experience autonomy frustration at a lower level than those in Profiles 2, 3, 5 and 7. The highest level of autonomy frustration was discovered among adolescents in Profile 3, followed by Profile 2. While there was no significant difference between the respective scores of those in Profiles 2 and 3, they had significantly higher autonomy frustration scores compared to adolescents in Profiles 1 and 7. A consideration of the attachment styles observed in participants in Profiles 6 and 4 suggests the potential for secure parent attachment to assume a more significant role in the regulation of autonomy needs among adolescents.

In terms of relatedness frustration scores, Profile 6 was followed by Profile 7 and 1; adolescents in these profiles seem to experience relatedness frustration at a lower level than those in Profiles 2, 3 and 4. The highest level of relatedness frustration was discovered among adolescents in Profile 2, followed by Profile 3. While there was no significant difference between the respective scores of those in Profiles 2 and 3, they had significantly higher relatedness frustration scores compared to adolescents in Profiles 5. Moreover, the respective scores of adolescents in Profile 2 were significantly higher than those in Profile 4. Overall, it is prudent to propose that frustration of relatedness needs during adolescence is closely linked with the dynamics of insecure peer attachment.

With respect to competence frustration scores, participants Profile 6 had significantly lower scores compared to those in the other profiles. Adolescents in Profile 3 exhibited the most pronounced competence frustration, followed by those in Profile 2. Pairwise comparisons revealed that the competence frustration scores of adolescents in Profile 3 significantly differed from those in the other profiles, except Profile 2. Moreover, those in Profile 2 had significantly higher competence scores compared to their counterparts in Profiles 4, 6, and 7. These findings imply that insecure parent and peer attachment may emerge as an important risk factor in the frustration of competence needs among adolescents.

In the last phase of the analysis, we also found significant differences between participants’ relative deprivation scores (school, family, and economic) by attachment profiles. Overall, adolescents in Profile 6 exhibited the lowest relative deprivation compared to all other profiles, followed by those in Profile 4. Participants in Profile 4 reported significantly lower total and family-related deprivation scores than all remaining profiles except Profile 6. This finding, therefore, suggests that adolescents reporting more secure parent attachment are likely to have a lower perception of relative deprivation when compared to their counterparts in the other profiles. Similarly, participants in Profile 7 demonstrated significantly lower total relative deprivation and family-related scores than those in Profiles 1, 2, 3, and 5. Yet, we found that participants in Profile 3 scored significantly higher on relative deprivation and its family subscale than the other profiles, underscoring that adolescents with insecure parent and peer attachment are likely to feel more disadvantaged in their interpersonal relationships than those exhibiting other attachment styles. When examined in terms of economic relative deprivation, Profile 6 demonstrated the lowest scores, followed by Profiles 4 and 7. Adolescents in these profiles seem to experience economic relative deprivation at a lower level than those in Profiles 1, 2, 3, and 5. Also, participants in Profile 3 reported the highest levels of economic relative deprivation, scoring significantly higher than those in Profiles 1, 2, and 5. Besides, pairwise comparisons of participants’ scores on the relative deprivation school subscale uncovered that only adolescents in Profile 6 perceived significantly lower relative deprivation in the school context than participants in the other profiles. Thus, it can be assumed that adolescents with secure parent and peer attachment are likely to perceive themselves as having a more favorable standing in their relationships in the school than those with other attachment styles.

## Discussion

The principal endeavor of this study was to explore the latent profiles of parent and peer attachment in a sample of Turkish adolescents through a person-centered approach and examine if participating adolescents falling within diverse attachment profiles exhibit significant differences in the indicators of life satisfaction, resilient mindset, BPNS/F, and relative deprivation. A growing body of research has adopted a person-centered approach to examine multiple attachment styles in adolescence, and these studies collectively contribute to our understanding of attachment in this developmental period (Flykt et al., 2021; Gao et al., 2024; He et al., 2018; Russotti et al., 2023; Withers, 2020; Yang and McGinley, 2022). Based on a thorough examination of the extant literature and to the best of our knowledge, the current research employs the largest sample size of any study that has used a person-centered approach to examine adolescent attachment and is the first to have been conducted with a Turkish sample. Moreover, our study offers a unique approach by comparing adolescent attachment profiles with diverse indicators oriented to adolescent well-being (e.g., resilient mindset, BPNS/F, and relative deprivation) rather than variables utilized in previous research. Hence, it has the potential to expand our current knowledge to provide novel insights into the intricate nature of the attachment hierarchy.

Our analysis identified seven distinct latent profiles of parent and peer attachment during adolescence, representing the highest number of profiles reported in previous research employing a person-centered methodology. Moreover, the profiles identified in this study are theoretically significant and bear some overlap with previous reports (Flykt et al., 2021; Gao et al., 2024; He et al., 2018; Russotti et al., 2023; Qu et al., 2025). These findings suggest that attachment hierarchies during adolescence may exhibit similar patterns across different countries and cultures and point to the potential universality of developmental aspects of adolescent attachment. At the same time, it is noteworthy that some of the profiles identified in this study were not reported in previous research, which underscores the likelihood that patterns of parent and peer attachment during adolescence may vary across samples and socio-cultural contexts.

The emergence of seven distinct latent profiles in the Turkish sample can be attributed to multiple factors. Türkiye’s transitional cultural dynamics, characterized by the coexistence of collectivist and individualist values (Kagitcibasi, 2005), may facilitate adolescents’ simultaneous maintenance of parental bonds and pursuit of autonomy. In addition, the traditional family structure may lead adolescents to have a differentiated perception of their relationships with parents, as mothers assume a more caregiving role while fathers tend to occupy comparatively distant and authoritative positions in this type of family structure. Furthermore, the use of a heterogeneous and highly representative sample in this study may have enabled the detection of latent profiles that, while potentially existing in the population, had not been previously identified in single studies. Linking each of these latent profiles to theoretical frameworks and examining their culturally specific implications is likely to yield valuable insights into the literature on adolescent attachment.

The majority of the sample was classified into one of the identified attachment profiles, with Profile 6 (*Secure Attachment to Mother, Father, and Peers*) representing the largest group, characterized by secure attachment across all relationships. The distribution of this profile within the sample aligns with meta-analytic evidence indicating that secure attachment styles generally occur more frequently than insecure attachment styles (Bakermans-Kranenburg and van IJzendoorn, 2009). Moreover, this finding aligns with the “universality” and “normativity” hypotheses of attachment theory (van Ijzendoorn and Sagi-Schwartz, 2008). While participants exhibiting insecure attachment patterns in all relationships were identified as part of Profile 3 (*Insecure Attachment to Mother, Father, and Peers*), those demonstrating secure attachment to their parents except their peers were classified as part of Profile 4 (*Secure Attachment to Mother and Father but Insecure Attachment to Peers*). As these three latent profiles were previously reported by Flykt et al. (2021) and He et al. (2018), our findings suggest that adolescent attachment hierarchies may follow overlapping cross-cultural and cross-regional patterns.

Profile 1 (*Predominantly Insecure Attachment to the Father*) and Profile 5 (*Predominantly Insecure Attachment to the Mother*) emerged as a consequence of adolescents’ distinct perceptions of their attachment relationships with their mothers and fathers. The attributes in these profiles underscore the notion that attachment to the mother and the father may not always follow the same trajectory concurrently and may, in fact, exhibit divergence. Consistent with our findings, Russotti et al. (2023) identified two distinct attachment profiles among a sample of female adolescents aged 15–19 years. These profiles characterize adolescents as either securely attached to their mothers and insecurely attached to their fathers or vice versa. In contrast, Flykt et al. (2021) focused on a sample of adolescents aged 17–19 years and surprisingly discovered a similar pattern between participants’ attachment to their mothers and fathers. The authors explained their results through the developmental transformation process where people perceive their relationships with their mothers and fathers more holistically, rather than separately, during late adolescence. Moreover, the literature highlights that mothers and fathers fulfill distinct yet complementary roles in the attachment process (Thompson, 1999). As an illustration, while mothers are often regarded as stable pillars, fathers are considered instrumental to the child’s exploration of the world (Dumont and Paquette, 2013; Grossmann et al., 2002; Keizer et al., 2019). These roles may prove effective in assessing adolescents’ attachment relationships with their parents. Indeed, adolescents perceiving complementary attachment roles in their parents may exhibit a more integrated evaluation style; yet, those encountering inconsistencies in their relationships with their parents may demonstrate a tendency toward a more fragmented evaluation. Adolescents’ more differentiated judgments may be influenced by a range of factors, including family system dynamics (e.g., coalition, spillover, and crossover effects), parent-specific characteristics (e.g., personality traits, psychopathology), dysfunctional parenting practices (e.g., neglectful, inconsistent, or authoritarian behaviors), and family structure (e.g., divorced parents). Future research may further develop our understanding of attachment by focusing on adolescents’ relationships with their mothers and fathers separately to uncover if attachment to mothers and fathers during adolescence is joint or fragmented. Moreover, investigating whether fragmented attachment has the potential to transform into a more joint attachment over time will contribute to expanding the findings of research with a person-centered approach.

Our analysis resulted in two additional attachment profiles, Profile 7 (*Low Parental Distance and Secure Peer Attachment*) and Profile 2 (*High Parental Distance and Insecure Peer Attachment*). The distinction between these two profiles was evident when examining the variations in levels of trust, communication patterns, and the extent of alienation from parents, as well as the attachment styles exhibited toward peers (secure-insecure). A closer examination of the attachment indicators in Profile 7 (*Low Parental Distance and Secure Peer Attachment*) shows that this profile included adolescents who (1) maintain beliefs in their parents’ capacity to provide care, security, and protection, (2) encounter challenges in effective communication with their parents, and (3) experience feelings of isolation/alienation from their parents but maintains a secure parent attachment. In this study, while we conceptualized the relational pattern involving parents as low parental distance, the pattern concerning peer relationships was characterized as secure peer attachment. When considered together, these patterns suggest that young individuals may prioritize a close friend as an attachment figure when their relationships with parents do not fully satisfy their relational needs. This attachment pattern has also been identified in a recent person-centered study by Qu et al. (2025). The authors labeled the profile as indicative of weak parent–child communication. Consistent with our findings, the researchers also reported that while struggling to communicate with their parents, approximately one in five adolescents exhibited moderate levels of secure peer attachment. Empirically supported, this attachment pattern can be interpreted in the context of developmental changes specific to adolescence and socio-cultural context. Given the significance of developmental tasks (e.g., achieving autonomy and forging new and more mature relationships with peers) during adolescence (Santrock, 2017; Steinberg, 2007), it is feasible to interpret the attachment patterns to parents in these profiles in relation to adolescence-specific changes. Changes in the cognitive domain, rising roles of peers, and increasing individuation from parents may result in alterations in attachment during adolescence (Buist et al., 2002; Hay and Ashman, 2003; Steinberg, 2007). The pursuit of autonomy, a hallmark of adolescent development, fosters an inclination to establish independence from parents and forge peer relationships (Steinberg, 2007; Santrock, 2017). Simultaneously, strengthened bonds with close friends can serve to differentiate how adolescents maintain relationships with their parents (Kobak et al., 2007; Rosenthal and Kobak, 2010). The literature emphasizes that separation-individuation is a complex process with the dynamics of trust and distance in adolescent–parent relationships (Koepke and Denissen, 2012). Due to the pursuit of autonomy and the emergence of conflict with parental figures in early and middle adolescence, adolescents temporarily disengage from their parents, perceiving them as worthless (Yaban, 2017). This dynamic explains the phenomenon where adolescents maintain trust in their parents while experiencing a sense of isolation from them. In addition to developmental changes during adolescence, the attachment pattern to parents mothers in this profile can also be interpreted within the socio-cultural context of the traditional Turkish family, where authoritarian parenting practices are prevalent (Yavuzer, 2016; Yavuzer, 2017), and in light of findings from studies on the value of children spanning about three decades (Kagitcibasi and Ataca, 2005; Kağıtçıbaşı, 2012). Authoritarian parenting is characterized by strict control over children’s behavior, the enforcement of rigid and inflexible rules, and expectations that children comply unquestioningly with parental directives. Consistent with elements of this parenting style, the said research in Türkiye identified that parents’ desired qualities in children often include “being a good person,” “achieving success,” “popularity,” “obedience,” and “independence” (Kagitcibasi, 2005) and that mothers particularly emphasize relational values (e.g., kindness, respect, and compliance) when defining the concept of a “good child” (Kağıtçıbaşı, 2012). Adolescents raised within this socio-cultural context may perceive their parents as reliable and responsible figures but still experience difficulties in communication and emotional distance in their relationships with them. It is then plausible that adolescents turn to peers to meet their needs for emotional intimacy, understanding, and sharing. Supporting this view, the literature indicates that when parents are less accessible due to limited availability or responsiveness, adolescents may seek secure attachment to a close friend as a haven or secure base under conditions of low risk or moderate challenges (Kobak et al., 2007). Thus, this profile may reflect relational structures specific to typical adolescent development.

While the relationship patterns with parents in Profile 2 are structurally similar to those observed in Profile 7, they differ in key respects: adolescents in Profile 2 exhibit lower levels of trust in their parents, higher levels of communication problems, and greater emotional distancing. This pattern suggests that increasing communication difficulties or conflicts with parents during adolescence may erode trust and contribute to greater alienation. Adolescents in this profile may experience moderate trust toward both parents while simultaneously encountering communication difficulties and shared experiences of alienation, which implies a family climate dominated by relational and communicative challenges. The escalation of loneliness in the context of impaired family communication may further contribute to the emergence of insecure attachment. The literature also highlights that communication problems in parent–adolescent relationships can undermine relational trust and compromise the secure attachment cycle (Kobak et al., 2015; Micucci, 2009). This profile also diverges from Profile 7 in terms of peer attachment indicators. Adolescents in Profile 2 not only exhibit pronounced relational distancing from their parents but also display insecure peer attachment, which may lead them to be more psychosocially vulnerable and increase their overall susceptibility to stressors.

On the other hand, we found significant differences between participants’ life satisfaction, resilient mindset, relative deprivation, and BPNS/F scores by these seven attachment profiles. Adolescents in Profile 6 (*Secure Attachment to Mother, Father, and Peers*) were the most advantaged subgroup regarding all mentioned outcome variables, which is consistent with the “Competence Hypothesis” proposing that secure attachment is associated with more positive outcomes (van Ijzendoorn and Sagi-Schwartz, 2008). Participants in this profile exhibited higher life satisfaction, enhanced capacity to cope with challenging experiences and unpredictable events, and increased BPNS (autonomy, relatedness, competence). However, they had lower BPNF and relative deprivation (school, family, economic). Overlapping with these findings, previous research indicated that adolescents perceiving their parents as a secure base and a source of support and forming secure peer attachment are likely to exhibit enhanced psychological well-being compared to their peers (Kobak et al., 2007; McElhaney et al., 2009). Our results are further substantiated by research reporting that secure attachment serves as a pivotal indicator of adolescents’ healthy development by enhancing their psychosocial adjustment (Armsden and Greenberg, 1987; Flykt et al., 2021; Greenberg et al., 1983; He et al., 2018; Laible, 2007; Laible et al., 2000). Moreover, our findings underscore the pivotal role of fostering secure attachments with multiple figures during adolescence based on the “Additive Hypothesis” (Dagan and Sagi-Schwartz, 2021), which proposes that secure attachments with multiple individuals during infancy foster more favorable developmental outcomes in adolescence.

Adolescents in Profile 4 (*Secure Attachment to Mother and Father but Insecure Attachment to Peers*) remained one step behind those in Profile 6 by life satisfaction, resilient mindset, autonomy satisfaction and frustration, competence satisfaction and frustration, and family and economic deprivation, but they maintained a relatively advantageous position compared to those in the other profiles. Replicating the conclusions of Greenberg et al. (1983), our findings showed that secure parent attachment during adolescence may be comparatively more salient than secure peer attachment in the mentioned domains (e.g., life satisfaction). Similarly, the relevant literature corroborates the notion that well-being indicators such as BPNS (Mikulincer and Shaver, 2015), personal autonomy (He et al., 2018), subjective well-being (Greenberg et al., 1983), and resilience (Darling Rasmussen et al., 2019) are highly associated with secure parent attachment. It was also emphasized that parent attachment during adolescence is important for optimal development (Moretti and Peled, 2004). In this context, we could reveal the protective role of secure parent and peer attachment during adolescence, partially overlapping with the literature. Nevertheless, adolescents in Profile 7 (*Low Parental Distance and Secure Peer Attachment*) had lower relatedness satisfaction and frustration and school deprivation scores when compared to those in the Profile 6 profile but outperformed their peers in the remaining profiles in these variables. Thus, the role of secure peer attachment should be recognized in adolescents’ relatedness needs satisfaction and perceived relative deprivation based on social comparisons regarding school life. Given the key role of school life in shaping adolescents’ academic, emotional, and social development (Crosnoe and Benner, 2015), secure peer attachment should also be considered for fostering a sense of relatedness and reducing perceived relative deprivation in the school setting. Moreover, this finding lends further support to the notion that peers emerge as a primary source of relatedness satisfaction during adolescence (Hay and Ashman, 2003). Another noteworthy observation in this study is that participants in these profiles had similar scores from the outcome variables measured, suggesting a convergence in their characteristics. This finding aligns with previous findings uncovering that adolescents who fail to establish sufficiently satisfying attachment relationships with their parents may endeavor to compensate for this deficiency by cultivating stronger ties with their peers (Flykt et al., 2021; Mayseless et al., 1998).

Adolescents in Profile 1 (*Predominantly Insecure Attachment to the Father*) and Profile (*Predominantly Insecure Attachment to the Mother*) also had almost overlapping scores on the outcome variables measured. It should be noted that participants in Profile 1 were more advantageous in these variables than their counterparts in Profile 5, although there were no significant differences between their scores except for relative economic deprivation. Considering that adolescents in Profile 1 were found to be securely attached to their mothers, our finding partially supports the notion that mothers maintain a significant role in adolescent attachment hierarchies (Fraley and Davis, 1997; Markiewicz et al., 2006). Nevertheless, our findings suggest that, irrespective of whether it is the mother or the father, predominant insecure parent attachment may yield comparable outcomes in adolescents’ life satisfaction, psychological resilience, satisfaction and frustration of basic psychological needs, and perceptions of relative deprivation. According to the horizontal hypothesis, one of the predictions derived from holistic models of attachment networks, the impact of attachment relationships in infancy on developmental outcomes is independent of the parent’s role. From this perspective, a child’s secure attachment to a single parent is expected to confer similarly positive developmental outcomes (Dagan and Sagi-Schwartz, 2021). In line with this prediction, the comparable mean scores among adolescents predominantly insecurely attached to either parent across the measured variables appear plausible. These findings underscore the potential protective function of secure parent attachment during adolescence. However, given that there is no full consensus in the literature regarding the validity of the horizontal hypothesis, it is advisable to interpret the findings of the current study cautiously.

We noted lower life satisfaction, resilient mindset, and BPNS scores among participants in Profile 2 (*High Parental Distance and Insecure Peer Attachment*) and Profile 3 (*Insecure Attachment to Mother, Father, and Peers*) compared to those in the other profiles. Adolescents in these profiles also demonstrated greater BPNF (autonomy, relatedness, competence) and perceived relative deprivation (school, family, and economic). Those in Profile 2 exhibited the lowest relatedness satisfaction and the highest relatedness frustration, emphasizing the key role of peers in relatedness satisfaction during adolescence. As expected, adolescents in Profile 3 exhibited more pronounced negative outcomes than those in the other profiles in terms of all other variables. Previous person-centered research also showed that low-quality attachment to parents and peers is linked with undesirable outcomes such as health problems and risk-taking behaviors (Flykt et al., 2021), anxiety, depression, problematic internet use (Gao et al., 2024), life dissatisfaction, and psychological distress (He et al., 2018). A distinct contribution of this study to the existing literature, unlike previous research, is its ability to reveal that Profile 3 represents a significant risk for autonomy satisfaction, competence satisfaction, resilient mindset and perceived relative deprivation among adolescents. Moreover, the potentially vulnerable and at-risk profile across the measured variables (Profile 3) suggests that the additive effect hypothesis may also be applicable during adolescence. In this regard, our findings extend the original assumption of the hypothesis that insecure attachment to both parents in infancy predicts the lowest levels of adaptive functioning (Dagan and Sagi-Schwartz, 2021). In adolescence, potential risks may not only arise from insecure attachment to both parents but may also be associated with the additional presence of insecure peer attachment.

This study is not free of a few limitations. Firstly, the employment of a cross-sectional design precluded the assessment of adolescents’ attachment profiles over time. Future research adopting a longitudinal design could yield more comprehensive insights into the developmental trajectories of attachment in adolescence. Secondly, the findings are limited to the constructs measured by the instruments utilized in this study. For example, we explored the attachment profiles of participating adolescents using the IPPA. Despite being a widely utilized tool to assess parent and peer attachment in adolescence, this instrument does not assess dimensions of insecure attachment (e.g., anxiety or avoidance). Prospective studies would benefit from integrating multi-method approaches (e.g., observations, interviews) in a mixed-method design. Scrutinizing the subject with the aid of qualitative data would enhance the interpretation of trust-distrust in the attachment profiles obtained through LPA. Thirdly, our results are predicated on adolescent reports, which may be subject to social desirability bias and compromise internal validity. Hence, future research may seek to support the adolescent perspective by soliciting the views of parents and peers. The fourth limitation pertains to the analytical method employed. While LPA enabled us to classify adolescents into distinct subgroups, factors such as sample size, sample characteristics, and the validity and reliability of the measures may have influenced determining the number of profiles. Moreover, due to the probabilistic nature of LPA, participants are assigned to profiles based on likelihood rather than absolute certainty, which may introduce ambiguity, particularly for individuals assigned near profile boundaries. To mitigate methodological limitations, we employed a multi-stage cluster sampling to achieve a large, heterogeneous sample. In addition, prior to conducting LPA, we performed analyses to assess the validity and reliability of the measures to minimize the risk of measurement-related limitations. Nonetheless, it should be noted that, due to the inherent nature of LPA, the profiles obtained may not necessarily replicate identically across different samples or cultural contexts. Finally, the sample consisted of Ankara-based high school students aged 14–18 years. The replication of this study in different countries, cultures, and sociodemographic contexts is recommended to provide more robust evidence on the generalizability or specificity of our findings.

Despite these limitations, we believe that our findings warrant scholarly interest. Parents and peers represent two fundamental environmental microsystems that play significant roles in adolescent development through reciprocal interaction during adolescence (Bronfenbrenner, 2005). In this regard, we concentrated on parent and peer attachment, identified seven distinct attachment profiles in a large, representative sample, and concluded that attachment does not always co-occur in adolescence. Moreover, we explored participating adolescents’ life satisfaction, resilient mindset, BPNS/F, and relative deprivation by the identified attachment profiles and established that the generated profiles offer not only statistical but also theoretical and practical validity. The findings indicate that secure parent and peer attachment is optimal for adolescent development, whereas the inverse scenario represents the least favorable outcome. Furthermore, our evidence indicates secure parent and peer attachment provides a protective effect on the psychological outcomes of adolescents; however, communication problems with parents and feelings of isolation can be partially compensated by secure peer attachment. In addition, predominant insecure parent/peer attachment also poses risks in terms of outcome variables. In a nutshell, this person-centered study attempted to elucidate the intricate nature of attachment hierarchies during adolescence, and our findings suggest that the development of targeted, customized strategies for adolescents with different attachment profiles is necessary while promoting their optimal development with attachment-based approaches.

## Data Availability

The datasets presented in this article are not readily available due to privacy and ethical restrictions. Requests to access the datasets should be directed to raziye.yuksel@hacettepe.edu.tr.
